# Disinhibitory circuit mediated by connections from vasoactive intestinal polypeptide to somatostatin interneurons underlies the paradoxical decrease in spike synchrony with increased border ownership selective neuron firing rate

**DOI:** 10.3389/fncom.2022.988715

**Published:** 2022-11-04

**Authors:** Nobuhiko Wagatsuma, Haruka Shimomura, Sou Nobukawa

**Affiliations:** ^1^Department of Information Science, Faculty of Science, Toho University, Funabashi, Japan; ^2^Department of Computer Science, Chiba Institute of Technology, Narashino, Japan; ^3^Department of Preventive Intervention for Psychiatric Disorders, National Center of Neurology and Psychiatry, Kodaira, Japan

**Keywords:** border ownership, selective attention, inhibitory interneuron subtypes, synchrony, disinhibition, computational model, visual cortices, figure-ground segregation

## Abstract

The activity of border ownership selective (BOS) neurons in intermediate-level visual areas indicates which side of a contour owns a border relative to its classical receptive field and provides a fundamental component of figure-ground segregation. A physiological study reported that selective attention facilitates the activity of BOS neurons with a consistent border ownership preference, defined as two neurons tuned to respond to the same visual object. However, spike synchrony between this pair is significantly suppressed by selective attention. These neurophysiological findings are derived from a biologically-plausible microcircuit model consisting of spiking neurons including two subtypes of inhibitory interneurons, somatostatin (SOM) and vasoactive intestinal polypeptide (VIP) interneurons, and excitatory BOS model neurons. In our proposed model, BOS neurons and SOM interneurons cooperate and interact with each other. VIP interneurons not only suppress SOM interneuron responses but also are activated by feedback signals mediating selective attention, which leads to disinhibition of BOS neurons when they are directing selective attention toward an object. Our results suggest that disinhibition arising from the synaptic connections from VIP to SOM interneurons plays a critical role in attentional modulation of neurons in intermediate-level visual areas.

## Introduction

**FIGURE 1:**
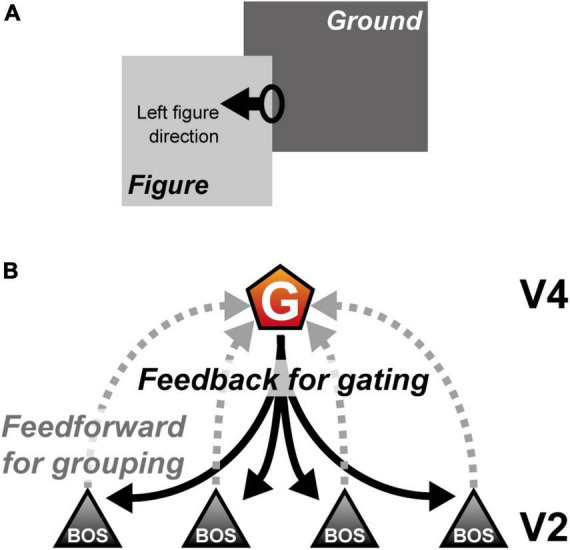
Border ownership and role of grouping (G-) cells. **(A)** Example of figure-ground segregation. In this illustration, the light gray “Figure” object seems to be in front of the dark gray “Ground” object. The figure direction from the oval drawn on the right contour of the “Figure” object is left. In this example, the left side of a contour owns the border (border ownership left). **(B)** The mechanism of a G-cell and border ownership selective (BOS) neurons (Craft et al., 2007; Mihalas et al., 2011). The activity of BOS neurons depends on the direction of the figure with respect to the border projected onto their classical receptive field, which is integrated by the G-cell to represent the grouping structure (gray dashed lines). Furthermore, the G-cell provides common feedback signals to V2 (black solid lines). For simplicity, this computational study did not include the mechanism by which the G-cells integrate BOS neuron activity, as shown by gray dashed lines.

The most fundamental but essential process for detecting a target location and perceiving a visual object is segregation of the figural region from the background in the visual scene (figure-ground segregation). In the nervous system, figure-ground segregation begins in early to intermediate visual cortical areas by determining the figure direction from the object contour (Lamme, 1995; Sajda and Finkel, 1995; Figure 1A). Physiological studies have reported that a majority of extrastriate (V2) neurons selectively respond to border ownership; neuron activity depends on the direction of the figure with respect to the border projected onto the classical receptive field (CRF) (border ownership selective (BOS) neurons; Zhou et al., 2000). To understand the neural mechanisms of figure-ground segregation and object recognition, various studies have examined the characteristics of BOS neurons using physiological, psychophysical, and computational methods (Sakai and Nishimura, 2006; Craft et al., 2007; Qiu et al., 2007; Sugihara et al., 2007, 2011; Dong et al., 2008; O’Herron and von der Heydt, 2009; Zhang and von der Heydt, 2010; Mihalas et al., 2011; Sakai et al., 2012; von der Heydt, 2015; Hu and Niebur, 2017; Wagatsuma and Sakai, 2017; Hu et al., 2019). According to these previous studies, BOS neurons may integrate feedforward inputs originating from visual stimuli with feedback signals from higher visual areas to represent the figure direction in the visual scene (Figure 1B). Selective attention mediated by feedback signals plays an essential role in determining and modulating BOS neuron activity in the V2 region.

**FIGURE 2:**
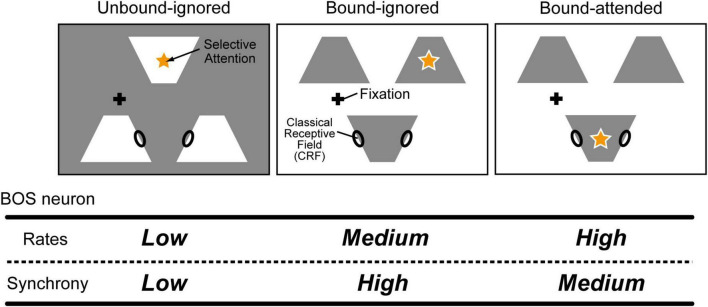
Example conditions for visual stimuli and attention in physiological experiments (modified from Martin and von der Heydt, 2015). Black ellipses on the edges of white or gray objects indicate the classical receptive fields (CRFs) for border ownership selective (BOS) neurons. In experiments, animals attended to one of the objects, illustrated by an orange star. The fixation point is illustrated as a black cross. Three separated white or gray objects were presented to animals. Modulation of BOS neurons with regard to their firing rates and spike synchrony is summarized in the bottom table. For the Unbound-ignored condition **(Left panel)**, the locations of the CRFs of two BOS neurons were allocated on the edges of two distinct white objects. In contrast, under the Bound-ignored condition, these two BOS neurons coded the figure direction of the same object. Under the Bound-attended condition, in addition to representations of the figure direction for the same object by two BOS neurons, animals directed their attention to this common object. These two BOS neurons preferentially responded when the stimuli shown in the bound conditions were presented. This pair of neurons representing the same object is referred to as a consistent pair. Selective attention further increased the firing rates of these neurons but decreased their spike synchrony (Martin and von der Heydt, 2015).

Selective attention is one of the most crucial functions in the brain for preferentially processing and perceiving the most important information at specific moments (Posner, 1980; Carrasco et al., 2004; Carrasco, 2011). Interestingly, selective attention not only modulates visual perception and neural responses for a variety of aspects (Ito et al., 1998; Lee et al., 1999; Mitchell et al., 2004) but also switches the perception of the figure direction and alters object recognition (Rubin, 2001). Interactions between visual stimuli and selective attention in BOS neurons in the V2 region are crucial for the establishment of neural representations of the figural region (Qiu et al., 2007; Martin and von der Heydt, 2015). In addition, computational studies using modeled BOS neurons have suggested that selective attention contributes to switching perception of the figure direction (Wagatsuma et al., 2008, 2013). However, selective attention can induce paradoxical modulation of BOS neuron activity between the firing frequency and spike synchrony; for example, attention increased the firing frequency of BOS neurons but decreased spike synchrony among BOS neurons during the coding of a common object (Martin and von der Heydt, 2015; bound conditions in Figure 2). This neurophysiological finding was reproduced by a simple network model with modulatory feedback signals mediated by N-methyl-D-aspartate (NMDA)-type synapses in addition to the feedforward inputs from α-amino-3-hydroxy-5-methyl-4-isoxazolepropionic acid (AMPA)-type synapses (Wagatsuma et al., 2016, 2021). However, these two previous models appeared to be oversimplified, especially because of the lack of inhibitory interneurons regulating the neuronal responses and dynamics.

Inhibitory interneurons play a critical role in flexibly regulating neuronal responses and dynamics by integrating feedforward inputs and feedback signals (Chen et al., 2017; Veit et al., 2017; Jang et al., 2020). Distinct subtypes of inhibitory interneurons expressing one of three genes, parvalbumin (PV), somatostatin (SOM), or vasoactive intestinal polypeptide (VIP), are critical members of the cortical microcircuit of the superficial layers in primary visual area V1 (Zhang et al., 2014; Neske et al., 2015; Mardinly et al., 2016; Cardin, 2018; Veit et al., 2021; Wagatsuma et al., 2022). In this network, feedback signals mediating selective attention are mainly projected to the VIP interneurons. Interestingly, the disinhibition arising from synaptic connections from VIP to SOM interneurons may underlie the attentional modulation of excitatory neurons because the SOM interneuron population is one of the main sources of inhibitory drive to the excitatory neuron population (Pfeffer et al., 2013; Zhang et al., 2014; Karnani et al., 2016; Mardinly et al., 2016; Yang et al., 2016; Lee et al., 2017, 2018; Dipoppa et al., 2018; Krabbe et al., 2019; Garrett et al., 2020; Millman et al., 2020; Gasselin et al., 2021). However, the role of such a disinhibitory network in modulating neuronal responses and spike synchrony remains largely unknown.

**FIGURE 3:**
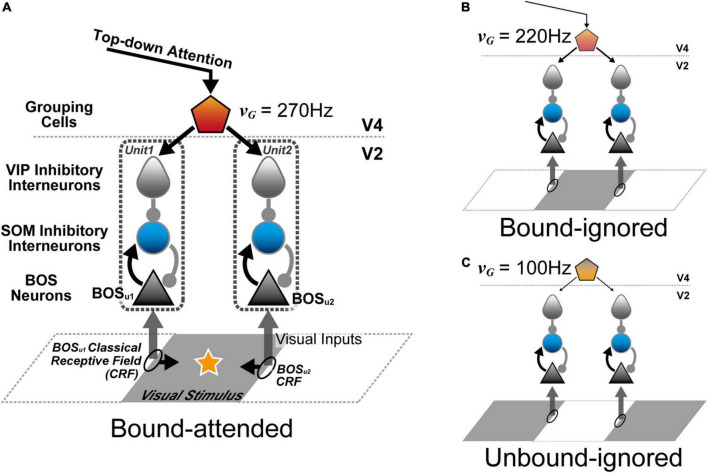
Architecture of the proposed microcircuit model for neural modulation of border ownership selective (BOS) neurons in V2. The V2 unit consisted of three types of spiking model neurons: an excitatory BOS neuron (black triangle) and somatostatin (SOM) (blue circle) and vasoactive intestinal polypeptide (VIP) interneurons (gray teardrop). Grouping (G-) cells (pentagon) in V4 projected the feedback signals, in addition to the representation of conditions for grouping structure and selective attention, to VIP model interneurons in the two V2 units. Black ellipses on the edges of the visual stimulus indicate the location of classical receptive fields of the V2 units. Black arrows from classical receptive fields (CRFs) represent the preferred direction of the figure for BOS neurons. Arrows with triangular and circular heads from model neurons show excitatory and inhibitory connections, respectively. **(A)** Stimulus configuration of the Bound-attended condition (Martin and von der Heydt, 2015). The classical receptive fields of these V2 units were projected onto the contours of the common visual stimulus. The star on the gray visual stimulus represents selective attention. In this condition, it was hypothesized that selective attention and a bound configuration of visual stimuli significantly activated G-cells (Wagatsuma et al., 2016, 2021), which was represented by the G-cell firing fate (ν*_*G*_*) of 270 Hz in the model simulations. **(B)** Stimulus configuration of the Bound-ignored condition. In this condition, the animal attended to a different object (not illustrated). Therefore, the visual stimulus was “ignored”. However, G-cells responded to the grouping structure of this gray object by integrating the BOS neuron activity in these units. To simulate this condition, ν*_*G*_* was set to 220 Hz. **(C)** Stimulus configuration of the Unbound-ignored condition. Under this condition, each V2 unit encoded different visual stimuli. This condition was presented by a ν*_*G*_* of 100 Hz for the simulations, the lowest rate among the three conditions. Note that we only varied the firing rates of G-cells (ν*_*G*_*) when simulating these three conditions. The visual stimuli projected onto the classical receptive fields were identical for all simulations. These inputs should be understood as originating from a population of neurons rather than from a single neuron.

In this study, to investigate the mechanism of attention-induced paradoxical modulation of BOS neurons in terms of their firing rates and spike synchrony, we developed a biologically-plausible microcircuit model consisting of spiking neurons including SOM and VIP interneurons in addition to excitatory BOS model neurons (Figure 3). Our proposed microcircuit model consisted of two V2 units with different CRF locations. BOS model neurons and SOM model interneurons interacted with each other in each V2 unit. In contrast, VIP model interneurons preferentially suppressed SOM interneuron responses. In addition, feedback signals mediating selective attention were projected to VIP interneurons in both V2 units, which induced disinhibition of BOS model neurons when directing selective attention toward an object. Simulations using our model exhibited a decrease in spike synchrony of BOS model neurons between the V2 units because of marked projection of feedback signals. However, a monotonic increase in the firing rate was found in BOS model neurons with increasing feedback signal frequency, which is consistent with the physiological findings (Martin and von der Heydt, 2015). These results suggested that disinhibition arising from the synaptic connections from VIP to SOM interneurons underlies BOS neuron modulation in the V2 region with respect to attention-induced enhancement of their firing rates and suppression of spike synchrony.

## Materials and methods

### Architecture of the proposed disinhibitory model

The architecture of the proposed microcircuit model is based on the grouping hypothesis (Figure 3; Craft et al., 2007; Mihalas et al., 2011; Russell et al., 2014; Wagatsuma et al., 2016, 2021; Wagatsuma, 2019). In these models, the grouping cell population (G-cells) in V4 not only integrates BOS neuron responses to represent the rough shape of objects but also modulates BOS neuron activity via feedback projections (Figure 1B). Our microcircuit model comprised two V2 units [Unit 1 (u1) and Unit 2 (u2)], each containing three neuron classes: excitatory BOS model neurons and SOM and VIP inhibitory model interneurons. These units represented the microcircuits in the V2 region that code border ownership with respect to the object edge projected onto their CRF. The locations of the CRFs of these units are shown as ellipses on squares in Figure 3. In our model, G-cells in V4 represented the grouping structure of the object, mediated selective attention, and provided feedback signals to two V2 units as common inputs.

Each V2 unit represented the basic processing unit for border ownership selectivity in the V2 region. These units included only the minimum number of model neurons and synaptic connections necessary to understand the fundamental mechanism underlying the modulation of BOS neurons observed in physiological experiments (Martin and von der Heydt, 2015); each unit consisted of one BOS, one SOM, and one VIP model neuron. Here, we assumed that the BOS model neuron in u1 (u2) preferentially responded to a right (left) figure direction from its CRF, which is represented as BOS_*u*1_ (BOS_*u*2_) (black arrows from ellipses in Figure 3A). Therefore, these BOS_*u*1_ and BOS_*u*2_ model neurons correspond to a consistent pair (Figure 2; Martin and von der Heydt, 2015; Wagatsuma et al., 2016). The arrows from model neurons in Figure 3A represent synaptic connections. Arrows with triangular and circular heads show excitatory and inhibitory connections, respectively. The structure of the V2 unit is based on previous computational studies (Buia and Tiesinga, 2008; Lee and Mihalas, 2017; Lee et al., 2017, 2018; Hertäg and Sprekeler, 2020; Keller et al., 2020; Wagatsuma et al., 2022). In our microcircuit model, the BOS neuron and SOM interneuron interact with each other within the unit. In contrast, in each unit, the VIP model interneuron locally inhibits the SOM model interneuron. In this local disinhibitory circuit, the sequential flow from the VIP to SOM interneuron within the unit plays a critical role in grouping-structure-induced and attention-induced modulation of BOS model neurons. Note that there are no direct inter-unit connections in our proposed model.

According to the grouping hypothesis (Craft et al., 2007; Mihalas et al., 2011; Wagatsuma et al., 2016, 2021), we hypothesized that G-cells not only encode the grouping structure of the presented object by merging BOS neuron activity but also mediate selective attention (Figure 1B). In addition, as shown by arrows representing the synaptic connections in Figure 3A, the feedback signals arising from G-cells are given as the common inputs to two V2 units, whereas feedforward inputs representing visual stimuli are independent processes. To investigate the mechanism of the attention-induced paradoxical modulation of physiological BOS neurons with regard to their firing rates and spike synchrony in detail, our current model did not include the activation of G-cells by BOS model neurons in response to the presented object, according to previous studies (Wagatsuma et al., 2016, 2021). Instead, G-cells were given as independent Poisson spike trains in this study. To simulate the “bound” conditions (Figure 3B), G-cells exhibited more activation than that in the “unbound” condition (Figure 3C). Note that under the “unbound” condition, BOS_*u*1_ and BOS_*u*2_ model neurons were not strongly activated because figure directions from the CRF of V2 units were inconsistent with the preference of BOS_*u*1_ and BOS_*u*2_ model neurons. Furthermore, in the Bound-attended condition (Figure 3A), selective attention to an object further increased the firing rate of the corresponding G-cell (ν*_*G*_*). In contrast, because the CRF content is identical irrespective of visual inputs and attention, the feedforward inputs had the same parameters (Figure 3). Therefore, in this study, the conditions for visual stimuli and attention were fully described by the firing frequencies of G-cells (Wagatsuma et al., 2016, 2021). We will further describe these points at section 2.3 “Numerical experiments”.

### Model neurons and synapses

In this study, we used integrate-and-fire neurons to describe BOS model neurons and all subtypes of inhibitory model interneurons (Buehlmann and Deco, 2008; Wagatsuma et al., 2016, 2021, 2022). The dynamics of the subthreshold membrane potential of a model BOS neuron (*V*_*BOS*_), SOM interneuron (*V*_*SOM*_), and VIP interneuron (*V*_*VIP*_) are given as follows:


(1) $$\frac{d\,V_{B\,O\,S}\,\left(t\right)}{d\,t}=-\frac{V_{B\,O\,S}\,\left(t\right)-E_{l}}{\tau_{m}^{B\,O\,S}}+\frac{I_{S\,O\,M}\,\left(t\right)+I_{V\,i\,s}\,\left(t\right)}{C_{m}}$$

(2) $$\frac{d\,V_{S\,O\,M}\,\left(t\right)}{d\,t}=-\frac{V_{S\,O\,M}\,\left(t\right)-E_{l}}{\tau_{m}^{S\,O\,M}}+\frac{I_{B\,O\,S}\,\left(t\right)+I_{V\,I\,P}\,\left(t\right)+I_{B\,G}\,\left(t\right)}{C_{m}}$$

(3) $$\frac{d\,V_{V\,I\,P}\,\left(t\right)}{d\,t}=-\frac{V_{V\,I\,P}\,\left(t\right)-E_{l}}{\tau_{m}^{V\,I\,P}}+\frac{I_{G}\,\left(t\right)+I_{B\,G}\,\left(t\right)}{C_{m}}$$

where $\tau_{m}^{B\,O\,S}$, $\tau_{m}^{S\,O\,M}$, and $\tau_{m}^{V\,I\,P}$ are the membrane time constants of excitatory BOS neurons and SOM and VIP inhibitory interneurons, respectively. *C*_*m*_ is the membrane capacitance. *E*_*l*_ indicates the leak-reversal potential. We summarized the neuronal model parameters of this study, which were chosen according to previous studies (Buehlmann and Deco, 2008; Neske et al., 2015; Lee et al., 2018), in Table 1. The spike threshold was *V*_*thr*_ = -50 mV and the membrane potential was reset to *V*_*reset*_ = -60 mV after spiking. *I_*BOS*_, I_*SOM*_*, and *I*_*VIP*_ represent the synaptic currents from the BOS neurons and SOM and VIP interneurons in the V2 unit, respectively. *I*_*BG*_, *I*_*Vis*_, and *I*_*G*_ indicate synaptic currents arising from external inputs based on background inputs, feedforward inputs arising from visual stimuli, and feedback signals from G-cells, respectively.

**TABLE 1:** Neuronal model parameters for border ownership selective (BOS) model neurons and somatostatin (SOM) and vasoactive intestinal polypeptide (VIP) model inhibitory interneurons.

		Parameter		
		
		BOS	SOM	VIP
***τ_*m*_***	Membrane time constant (ms)	10.5	11.8	10.9
***τ_*ref*_***	Refractory period (ms)	2.0	1.0	1.0
***C*** **_*m*_**	Membrane capacitance (pF)	200		
***E*** **_*l*_**	Leak reversal potential (mV)	–70		

In this study, BOS model neurons made intra-unit connections to SOM model interneurons (Figure 3). *I*_*BOS*_, representing the synaptic current from BOS neurons, was mediated by AMPA-type currents (Buehlmann and Deco, 2008; Deco and Thiele, 2011; Wagatsuma et al., 2016, 2021) and was defined as:


(4) $$I_{B\,O\,S}\,\left(t\right)=w_{\mathrm{BOS}}\cdot g_{\mathrm{BOS}}\,\left(V\,\left(t\right)-V_{E}\right)\,s_{\mathrm{BOS}}\,\left(t\right)$$

where *V*_*E*_ = 0 mV represents the reversal potential of BOS neurons and *V* is the subthreshold membrane potential of a model neuron (see also Eqs. 1–3). *s*_*BOS*_ indicates the fraction of open channels in a synapse from a BOS to a SOM model neuron. *g*_*BOS*_ is the conductance of the fully activated synapse, chosen as *g*_*BOS*_ = 0.64 for the connection from BOS to SOM model neurons (Lee et al., 2018). *w*_*BOS*_ = 70 was the weight parameter. Note that we applied the weight parameter *w* to all model synapses in the V2 units to regulate network activity because our network model consisted of only the minimum number of neurons and synaptic connections. These weight parameters are free in our model and were chosen to decrease the firing rates of BOS model neurons so that they are within a physiologically realistic range.

The fraction of open channels in a synapse from a BOS to a SOM model neuron (*s*_*BOS*_) was determined as follows:


(5) $$\frac{{\mathrm{ds}}_{\mathrm{BOS}}}{\mathrm{dt}}=-\frac{s_{\mathrm{BOS}}\,\left(t\right)}{\tau_{s\,y\,n}^{B\,O\,S}}+\sum_{k}\delta \,\left(t-t_{j}^{k}-d_{j}\right)$$

where the postsynaptic decay time constant was $\tau_{s\,y\,n}^{B\,O\,S}$ = 5.4 ms (Lee et al., 2018). The sum over *k* includes all spikes from connecting BOS neurons. Each spike was entered as a Dirac delta function, δ(*t*), assuming a non-zero value at the spike times of the visually driven input neurons ($t_{j}^{k}$) (zero elsewhere) and integrating to unity over any interval that included $t_{j}^{k}$. The delay from the BOS neuron was *d*_*j*_ = 2.0*ms*.

Synaptic currents from the two inhibitory interneuron subtypes reduced the membrane potentials of postsynaptic model neurons. Synaptic currents from inhibitory model interneurons *I*_*Inh*_ were given as follows:


(6) $$I_{I\,n\,h}\,\left(t\right)=w_{I}\cdot g_{I}\,\left(V\,\left(t\right)-V_{I}\right)\,s_{\mathrm{Inh}}\,\left(t\right)$$

where the subscript of *Inh* represents the inhibitory interneuron subtype, either SOM or VIP in this study. *V*_*I*_ = -70 mV is the reversal potential of the inhibitory interneurons. *g*_*I*_ represents the synaptic conductance of a fully open synapse of a specific subtype of inhibitory interneuron and depends on the classes of the presynaptic and postsynaptic neurons (Table 2; Hoffmann et al., 2015; Lee et al., 2018). We used the inhibitory weight parameter *w*_*I*_ to regulate the responses of the V2 units (Table 2). The fraction of open channels in a synapse of a SOM or VIP interneuron, *s*_*Inh*_, was given as follows:


(7) $$\frac{{\mathrm{ds}}_{\mathrm{Inh}}}{\mathrm{dt}}=-\frac{s_{\mathrm{Inh}}\,\left(t\right)}{\tau_{s\,y\,n}^{\mathrm{Inh}}}+\sum_{k}\delta \,\left(t-t_{j}^{k}-d_{j}\right)$$

**TABLE 2:** Parameters of postsynaptic currents including synaptic conductance, weight, and decay time constants depending on the classes of presynaptic and postsynaptic neurons.

	Intra-unit synaptic parameters			

		Parameters		
		
		BOS→SOM	SOM→BOS	VIP→SOM
***g***	Synaptic conductance (nS)	0.64	1.40	1.80
***w***	Weight parameter	70.0	788.0	612
***τ_*decay*_***	Synaptic-decay time constants (ms)	5.4	13.1	13.1

	**Synaptic parameters of external inputs**				

		**Parameters**			
		
		**Feedforward to BOS**	**Background to SOM**	**Background to VIP**	**Feedback to VIP**

***g***	Synaptic conductance (nS)	0.104	0.64	0.59	0.59
***w***	Weight parameter	36.4	14.0	5.6	22.4
***τ_*decay*_***	Synaptic-decay time constants (ms)	2.0	2.0	2.0	2.0
ν	Rates (Hz)	200.0	100.0	100.0	See main text

Note that inputs to the network should be understood as originating from a population of neurons rather than from a single neuron.

where $\tau_{s\,y\,n}^{\mathrm{Inh}}$ is the postsynaptic decay time constant, selected according to previous studies (Table 2; Pfeffer et al., 2013; Lee et al., 2018). As in the description of synaptic currents of BOS model neurons in Eq. 5, the sum over *k* is over the spike time ($t_{j}^{k}$); here, these were the times of spikes occurring in the SOM or VIP interneurons. The delay from the SOM and VIP interneurons was *d*_*j*_ = 1.0*ms*.

Recent studies have provided estimates of postsynaptic current parameters depending on the neuron class and subtype, such as synaptic conductances (*g*_*BOS*_ and *g*_*I*_) and decay time constants (τ_*BOS*_ and τ_*Inh*_) (Pfeffer et al., 2013; Hoffmann et al., 2015; Lee et al., 2017, 2018; Table 2) summarizes the details of the synaptic parameters for our network model.

*I*_*BG*_, *I*_*Vis*_, and *I*_*G*_ in Eqs. 1–3 represent the synaptic currents of background inputs, feedforward inputs, and feedback signals to model neurons. In this study, the V2 unit received input from two external sources: feedforward inputs representing visual stimuli and feedback signals from G-cells. Feedforward inputs originating from visual stimuli were independently projected onto BOS model neurons in V2 units. In contrast, VIP model interneurons in the two V2 units received common feedback signals from G-cells, which activated BOS model neurons by inhibiting SOM model interneurons. In addition, we provided background inputs to SOM and VIP interneurons to induce spontaneous activity. These external inputs to each model neuron were given as an independent Poisson spike train. For simplicity, these external inputs were mediated by an AMPA-type synapse and can be defined as follows (Buehlmann and Deco, 2008; Deco and Thiele, 2011; Wagatsuma et al., 2016, 2021).


(8) $$I_{I\,n\,p\,u\,t}\,\left(t\right)=w_{\mathrm{Input}}\cdot g_{\mathrm{Input}}\,\left(V\,\left(t\right)-V_{E}\right)\,s_{\mathrm{Input}}\,\left(t\right)$$

where the subscript *Input* represents the type of external input for background input, feedforward input, or feedback signals. *g*_*Input*_ is the conductance of the fully activated synapse for background input, feedforward input, or feedback signals, which was selected according to previous studies (Buehlmann and Deco, 2008; Wagatsuma et al., 2016; Lee et al., 2018). *W*_*Input*_ represents the weight parameter for V2 unit activity regulation. The fraction of open channels in model neurons induced by external inputs (*s*_*Input*_) was determined as follows:


(9) $$\frac{{\mathrm{ds}}_{\mathrm{Input}}}{\mathrm{dt}}=-\frac{s_{\mathrm{Input}}\,\left(t\right)}{\tau_{\mathrm{Input}}}+\sum_{k}\delta \,\left(t-t_{j}^{k}-d_{j}\right)$$

where the postsynaptic decay time constant for external inputs was τ_*Input*_ = 2.0 ms, irrespective of the class and subtype of the target neuron. See also Eqs. 5 and 7 for detailed descriptions of these equations. The delay from these excitatory external inputs was *d*_*j*_ = 2.0*ms*.

In this study, the firing rates of G-cells (ν*_*G*_*), which generate feedback signals to both V2 units, represent the grouping structure of visual stimuli and attentional conditions (Figure 3). In contrast, the background and feedforward input activity were fixed through all simulations, irrespective of conditions (Wagatsuma et al., 2016, 2021). Details of the frequencies of these external inputs are shown in the Numerical experiments section.

### Numerical experiments

In our model, we applied background inputs to SOM and VIP model interneurons in V2 units. These background inputs were given as independent 100-Hz Poisson spike trains. Furthermore, each BOS model neuron received feedforward inputs representing object borders. Because the CRF contents were identical for all visual stimuli used by Martin and von der Heydt (2015), in all simulation conditions, we modeled the feedforward inputs as Poisson spike trains with a mean rate of 200 Hz. These inputs should be understood as originating from a population of neurons rather than from a single neuron (Wagatsuma et al., 2016, 2021).

The G-cells generating the feedback signals were simulated by Poisson spike trains similar to other external inputs. G-cells are hypothesized to integrate BOS neuron responses to represent the grouping structure and rough shapes of objects in the scene (Craft et al., 2007; Wagatsuma et al., 2016, 2021). To examine the mechanism of BOS neuron response modulation, we increased the firing rates of G-cells under the bound conditions from that of the unbound condition (Figures 3B,C), in contrast to other external inputs. In the Unbound-ignored condition, G-cells fired with an average frequency of 100 Hz. If the object was present but not attended (Bound-ignored), the firing frequency was increased to 220 Hz, approximately doubling the rate of the Unbound-ignored condition. In addition, under the Bound-attended condition, the G-cells received both bottom-up inputs from BOS neurons and top-down signals from attentional control areas; therefore, the firing frequency of G-cells was further increased to 270 Hz (Figure 3A). We investigated how G-cell activation modulated BOS model neuron responses. Note that, similar to feedforward and background inputs, the feedback spike trains should be understood as activity originating from a population of G-cells.

According to previous studies (Wagatsuma et al., 2016, 2021), we integrated the differential equations using a fourth-order Runge-Kutta algorithm with a time step of 0.1 ms. We performed 50 model simulations for a length of 201 biological seconds per condition to assure the reproducibility of the model responses. In addition, we repeated this trial 10 times to average jitter-reduced synchrony (see also the Jitter method for tight synchrony section). The first second of the simulated results was always discarded to minimize the effect of transients. The code for the simulations was written in the C programming language.

### Analysis of spike synchrony of border ownership selective model neurons between V2 units

In this study, we computed the spike synchrony of BOS model neurons between V2 units according to the methods of previous studies (Martin and von der Heydt, 2015; Wagatsuma et al., 2016, 2021). Spike synchrony was computed by dividing time into 1-ms bins. There was either 0 or 1 spike per bin. To compute spike synchrony, we transformed a spike train into a binary vector $S_{j}^{i}\,\left(n\right)$, where *n* is the bin index, *i* is the trial number, and *j* is the identity of the neuron (*BOS_*u*1_* or *BOS_*u*2_*). The value 0 was assigned to each component of $S_{j}^{i}\,\left(n\right)$ if there was no spike between the interval *n* and *n* + *1* in the *BOS*_*j*_ model neuron during trial *i*. In contrast, the value 1 was given if a spike was present. To quantify spike synchrony, we computed the cross-correlation on the basis of the two spike trains, $S_{u\,1}^{i}\,\left(n\right)$ and $S_{u\,2}^{i}\,\left(n\right)$, of the *BOS_*u*1_* and *BOS_*u*2_* neurons (Martin and von der Heydt, 2015; Wagatsuma et al., 2016, 2021).

The cross-correlation function, *CC*^i^* (τ*), between two spike trains, $S_{u\,1}^{i}\,\left(n\right)$ and $S_{u\,2}^{i}\,\left(n\right)$, was computed as follows:


(10) $${\mathrm{CC}}^{i}\,\left(\tau \right)=\sum_{\mu =1000-\mathrm{wd}}^{201000+\mathrm{wd}}\left(S_{u\,1}^{i}\,\left(\mu +\tau \right)-f_{u\,1}^{i}\right)\,\left(S_{u\,2}^{i}\,\left(\mu \right)-f_{u\,2}^{i}\right)$$

(11) $$f_{j}^{i}=\frac{1}{\Theta }\,\sum_{n=1000}^{201000}S_{j}^{i}\,\left(n\right)$$

where *wd* = 250 ms is the maximal window of the cross-correlation function and τ is the time lag between the spike trains (*-wd* ≤ τ ≤ *wd*). $f_{j}^{i}$ is the mean spike count per bin of the spike train of *BOS*_*j*_ in trial *i*. Θ = 200 s in Eq. 11 is the length of trials in simulated biological seconds. Subtraction of $f_{j}^{i}$ from the neuron spike train in each trial was performed to compensate for the strength of spike synchrony depending on the modulation of firing rates, e.g., those produced by selective attention (Roelfsema et al., 2004).

The correlogram, CCG, was computed by averaging over all trials of CC, as follows:


(12) $$\mathrm{CCG}\,\left(\tau \right)=\frac{1}{\Theta }\,{\left\langle {\mathrm{CC}}^{i}\,\left(\tau \right)\right\rangle }_{i}$$

where ⟨ ⟩_*i*_ in Eq. 12 denotes the average over trials *i*. We smoothed correlograms using a Gaussian kernel with σ = 4 ms for comparisons with the neurophysiological results (Martin and von der Heydt, 2015).

The integral of the correlogram (Eq. 10) in the range between *-T* and +*T* represents the magnitude of BOS model neuron synchrony between V2 units:


(13) $$M^{i}=\sum_{\tau =-T}^{T}{\mathrm{CC}}^{i}\,\left(\tau \right)\times \mathrm{binsize}$$

where the bin size was set to 1 ms. The mean magnitude of synchrony over trials is given by


(14) $$\mathrm{AM}={\left\langle M^{i}\right\rangle }_{i}$$

“Loose synchrony” (correlations on the order of tens of milliseconds) was computed using *T* = *40* ms, according to previous studies (Wagatsuma et al., 2016, 2021).

### Examination of tight synchrony using the jitter method

Martin and von der Heydt (2015) applied jitter methods (Smith and Kohn, 2008; Amarasingham et al., 2012) to obtain physiological data from BOS neurons and investigate the characteristics of short timescale synchrony (tight synchrony). In addition, tight synchrony was used as an index to quantify the responses of computational models previously proposed (Wagatsuma et al., 2016, 2021). According to these previous studies, we also computed the tight synchrony of BOS model neurons between V2 units. In this section, we describe the essential characteristics of the jitter methods. For detailed full descriptions of the computation of tight synchrony, we refer the reader to previous studies (Martin and von der Heydt, 2015; Wagatsuma et al., 2016, 2021).

Jitter methods were applied to test the hypothesis that neurons operate at or below any specific temporal resolution (Smith and Kohn, 2008; Amarasingham et al., 2012). In this method, the data from each neuron was divided into bins based on the jitter window, starting at the stimulus onset. Each spike of each neuron was then independently moved to a new location within the jitter window, which was chosen uniformly at random from the jitter window in which it originally appeared (jittered spike train; see also Figure 3 in Amarasingham et al., 2012). In this manner, the spike count in each bin and the neuron’s poststimulus time histogram were preserved in the resampled data. Many correlations between jittered spike trains were computed (jittered correlation), and the mean was subtracted from the original correlation, resulting in the jitter-reduced correlation (tight correlation) (Wagatsuma et al., 2016, 2021). The advantage of this method is that it helps to disambiguate short and long temporal correlations in the correlograms. Interestingly, smaller jitter windows remove more of the long-timescale correlation between neurons (loose synchrony) while preserving the short-timescale correlation (tight synchrony). To test for tight synchrony, we generated 200 jittered spike trains with 20-ms jitter windows, producing an ensemble of surrogate spike trains from which a distribution of surrogate correlograms was calculated. The mean of this distribution was then subtracted from the correlograms of the original spike trains, as well as from each surrogate correlogram. This provided a jitter-derived correlogram and the confidence limits of the null hypothesis. Furthermore, similar to the case of loose synchrony, we also computed the integral of tight synchrony in the range between -5 and 5 ms.

## Results

**FIGURE 4:**
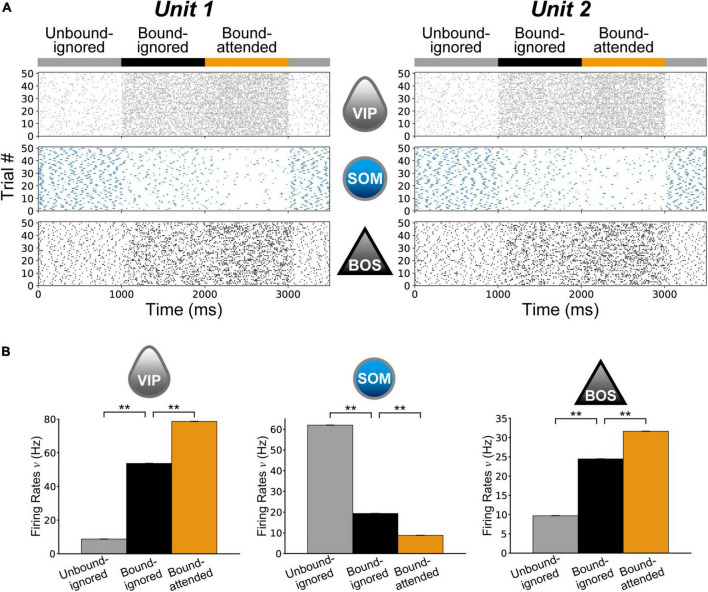
Neuronal responses in the proposed microcircuit model. **(A)** Raster plot showing 50 spike trains of model neurons for Unit 1 **(Right column)** and Unit 2 **(Left column)**. The gray **(Top)**, cyan **(Middle)**, and black **(Bottom)** dots represent vasoactive intestinal polypeptide (VIP), somatostatin (SOM), and border ownership selective (BOS) neuron spikes, respectively. For these plots, model simulations began with the Unbound-ignored condition with a ν*_*G*_* of 100 Hz (Figure 3C). The G-cell activity was increased from 1,000 to 2,000 ms to simulate the model under the Bound-ignored condition (Figure 3B). G-cells were further activated from 2,000 to 3,000 ms to represent the Bound-attended condition (Figure 3A). BOS_*u*1_ and BOS_*u*2_ model neurons were markedly activated with G-cell activation. **(B)** Mean firing rates of VIP **(Left)** and SOM **(Middle)** model interneurons and BOS model neurons **(Right)** during 10 repeated trials in 50 simulations. Gray, black, and orange bars represent the rates for the Unbound-ignored, Bound-ignored, and Bound-attended conditions, respectively. Error bars indicate the standard error, which was very small in these simulations. Firing rates of VIP model interneurons and BOS model neurons increased with G-cell activation. In contrast, the firing rate of SOM model interneurons exhibited a contrasting modulation pattern compared with those of the other model neuron classes. Asterisks indicate significant differences between conditions (**p < 0.01, *t*-test).

We performed numeric simulations of the proposed model with various conditions mimicking the experiments of Martin and von der Heydt (2015). Figure 4A demonstrates spike raster plots of BOS, SOM, and VIP model neurons in two V2 units for 50 simulation trials. For the raster plots, G-cells were activated in the Unbound-ignored condition between 0 and 1,000 ms, in the Bound-ignored condition between 1000 and 2,000 ms, and in the Bound-attended condition between 2,000 and 3,000 ms. BOS_*u*1_ and BOS_*u*2_ neurons were markedly activated with facilitation of the feedback signals arising from G-cells.

### Firing rates of model neurons comprising V2 units

First, we investigated the influence of G-cell activity levels on the firing rates of model neurons in V2 units. Figure 4B shows a summary of the average firing rates of VIP and SOM model interneurons and BOS model neurons for the Unbound-ignored (gray bars), Bound-ignored (black bars), and Bound-attended (orange bars) conditions. Firing rates of BOS neurons (bottom panel in Figure 4B) and VIP interneurons (top panel in Figure 4B) were significantly higher in the bound than in the unbound conditions (*t*-test, *p* < 0.01 for BOS neurons and *p* < 0.01 for VIP interneurons). In addition, the firing rates of these model neurons for the Bound-attended condition were significantly increased compared with those of the Bound-ignored condition (*t*-test, *p* < 0.01 for BOS neurons and *p* < 0.01 for VIP neuron). These grouping-structure-induced and attention-induced enhancements of BOS model neuron activity were consistent with the physiological results (Qiu et al., 2007; Martin and von der Heydt, 2015). In contrast, modulation of the firing rate pattern of SOM model interneurons was opposite to that of BOS model neurons and VIP model interneurons. These results imply that feedback signals arising from G-cells play a critical role in modulation of the spike frequency of neurons comprising V2 units.

### Spike synchrony of border ownership selective model neurons between V2 units

**FIGURE 5:**
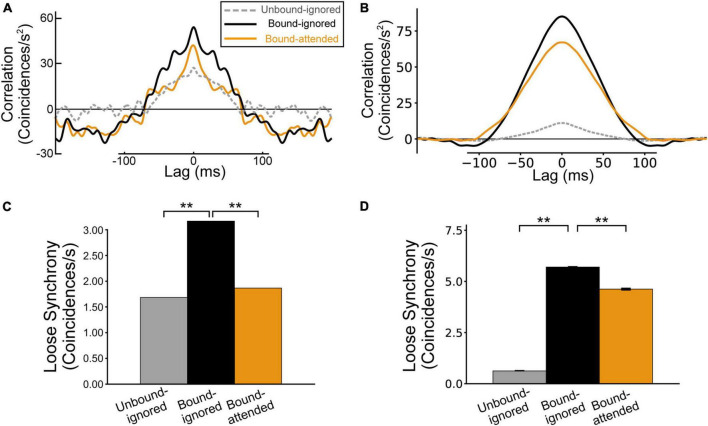
Spike synchrony of border ownership selective (BOS) model neurons between V2 units. The gray, black, and orange lines (bars) represent the spike correlation (loose synchrony) for the Unbound-ignored, Bound-ignored, and Bound-attended conditions, respectively. **(A)** Spike correlation for physiological BOS neurons, modified from Martin and von der Heydt, 2015. **(B)** Spike correlation between BOS model neurons in two different V2 units. The modulation patterns of model simulations were qualitatively consistent with the physiological results. **(C)** Loose synchrony observed in physiological BOS neurons (Martin and von der Heydt, 2015), which was computed by integrating the spike synchrony **(A)** between lags of -40 and + 40 ms (see also Eq. 11). **(D)** Simulation results for loose synchrony of BOS model neurons. The magnitude of loose synchrony for the Bound-ignored condition was significantly stronger than that of the other two conditions, which were quantitatively consistent with those of physiological observations (Martin and von der Heydt, 2015). Asterisks indicate significant differences between conditions (**p < 0.01, *t*-test). Error bars indicate the standard error.

A previous physiological study reported that, for pairs of BOS neurons with consistent border ownership preference, stimulation by a common object increased loose synchrony (correlations on the order of tens of milliseconds), whereas selective attention to the object decreased synchrony (Martin and von der Heydt, 2015; Figure 5A). To understand the mechanism underlying spike synchrony between BOS neurons, we performed simulations by computing spike train correlations of BOS model neurons between V2 units for the Unbound-ignored, Bound-ignored, and Bound-attended conditions. Loose correlations between BOS_*u*1_ and BOS_*u*2_ neurons for the Unbound-ignored, Bound-ignored, and Bound-attended conditions are demonstrated in Figure 5B. As described in the “Materials and methods” section, we smoothed these loose correlations using a Gaussian kernel with σ = 4 ms (Martin and von der Heydt, 2015). Loose correlations of VIP and SOM model interneurons between two V2 units are shown in Supplementary Figure 1. Marked loose correlation peaks were observed at a lag of zero for these three conditions. The correlation was markedly stronger for the Bound-ignored condition (black line in Figure 5B) than that for the Unbound-ignored condition (dashed gray line in Figure 5B). In contrast, the correlation for the Bound-attended condition (orange line in Figure 5B) was markedly lower than that for the Bound-ignored condition. The grouping-structure-induced increase and attention-induced decrease in loose correlation shown by our simulations were qualitatively consistent with the physiological observations (Figure 5A). Additionally, the widths of loose correlations were markedly distinct among neuron classes and subtypes (Figure 5B and Supplementary Figure 1), which might arise from differences in the postsynaptic decay time constant. We will further discuss this possibility in the Discussion section.

To statistically compare the spike synchrony of BOS model neurons among conditions, we computed the loose synchrony by integrating loose correlation in the range of ± 40 ms around a lag of zero (Figure 5C; Martin and von der Heydt, 2015; see “Materials and methods” section). Figure 5D summarizes the loose synchrony in our simulations. Loose synchrony for the Bound-ignored condition (black bar in Figure 5D) was significantly higher than that for the Unbound-ignored condition (gray bar in Figure 5D) (*t*-test, *p* < 0.01). In contrast, interestingly, a significant decrease was observed in the synchrony from the Bound-ignored condition (black bar in Figure 5D) to the Bound-attended condition (orange bar in Figure 5D) (*t*-test, *p* < 0.01). These simulation results correspond to the physiological findings for a consistent pair of BOS neurons (Martin and von der Heydt, 2015; Figures 5A,C).

### Tight synchrony of border ownership selective model neurons between V2 units

**FIGURE 6:**
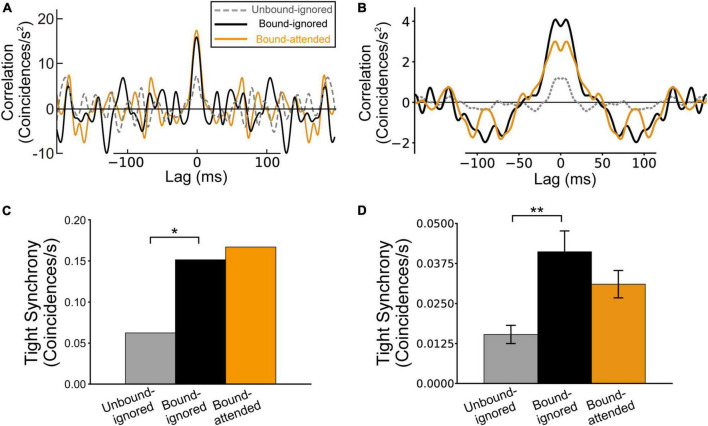
Tight synchrony between border ownership selective (BOS) neurons, which exhibited reduced spike synchrony after removing the correlation between jittered spike trains with a jitter window of Δ = 20 ms (see also Martin and von der Heydt, 2015; Wagatsuma et al., 2021). The gray, black, and orange lines (bars) indicate tight correlation (synchrony) for the Unbound-ignored, Bound-ignored, and Bound-attended conditions, respectively. **(A)** Normalized jitter-reduced correlation for physiological BOS neurons, modified from Martin and von der Heydt (2015) and Wagatsuma et al. (2021). **(B)** Normalized jitter-reduced correlation among BOS model neurons. A marked peak is present at a lag of 0 ms for the Bound conditions. **(C)** Tight synchrony observed in physiological BOS neurons, which was computed by integrating the jitter-reduced correlation **(A)** in the range between lags of-5 and + 5 ms (see also “Materials and methods” section). **(D)** Tight synchrony of BOS model neurons between V2 units. Asterisks indicate significant differences between conditions (*p < 0.05, **p < 0.01, *t*-test). Error bars represent the standard error.

In previous studies, tight synchrony (Smith and Kohn, 2008; Amarasingham et al., 2012) was used to characterize the responses of physiological and model BOS neurons (Martin and von der Heydt, 2015; Wagatsuma et al., 2016, 2021). Martin and von der Heydt (2015) reported significant peaks of tight synchrony around a lag of zero for consistent pairs of physiological BOS neurons in bound conditions but not in the unbound condition (Figure 6A). We applied the same analysis to our simulation results. For detailed full descriptions of the computation of tight synchrony, we refer the reader to previous studies (Martin and von der Heydt, 2015; Wagatsuma et al., 2016, 2021).

Jitter-reduced correlations (tight correlations) of BOS model neurons between V2 units for the Unbound-ignored, Bound-ignored, and Bound-attended conditions are summarized in Figure 6B. Despite the absence of common direct inputs to BOS_*u*1_ and BOS_*u*2_ model neurons, we observed a marked peak of tight correlation between BOS model neurons around a lag of zero for the Bound-ignored (black line in Figure 6B) and Bound-attended (orange line in Figure 6B) conditions. However, the tight correlation curves based on our simulation data seemed to be slightly broader than those observed in physiological experiments (Figure 6A; Martin and von der Heydt, 2015). In addition, we did not find tight correlation peaks at a lag of zero because jittered correlations around a lag of zero seemed to be sharper than those of loose correlations (Supplementary Figure 2).

Similar to the statistical comparison of loose synchrony in our simulation results, we computed tight synchrony by integrating jitter-reduced correlation in the range of a ± 5 ms interval around a lag of zero. Figures 6C,D summarize the magnitude of the tight synchrony for physiological (Martin and von der Heydt, 2015) and model BOS neurons, respectively. A significant difference was observed in the tight synchrony of our simulation results between the Unbound-ignored (gray bar in Figure 6D) and Bound-ignored conditions (black bar in Figure 6D) (*t*-test, *p* < 0.01). In contrast, the tight synchrony of BOS model neurons between V2 units slightly decreased from the Bound-ignored (black bar in Figure 6D) to the Bound-attended condition (orange bar in Figure 6D). However, we did not find a significant difference in this modulation of tight synchrony (*t*-test, *p* = *0.12*). These modulation patterns for tight synchrony shown by our model simulations were consistent with the physiological results for a consistent pair of BOS neurons (Martin and von der Heydt, 2015; Figure 6C). These results suggested that a disinhibitory network based on connections from VIP to SOM interneurons, at least in part, contributes to the grouping-structure-induced and attention-induced modulation of BOS neuron pair responses when representing the same object.

### Responses in border ownership selective model neurons as a function of the firing rates of G-cells in V4

Our proposed disinhibitory network model including two subtypes of inhibitory interneurons reproduced the physiologically observed characteristics of firing rate and spike synchrony modulation in BOS neurons (Martin and von der Heydt, 2015). To determine the mechanism by which the disinhibitory network modulates BOS model neuron activity, we performed simulations with the model while systematically varying the mean firing rates of G-cells from 0 to 350 Hz in steps of 10 Hz. In these simulations, the firing rate of feedforward inputs representing visual stimuli was fixed to 200 Hz.

**FIGURE 7:**
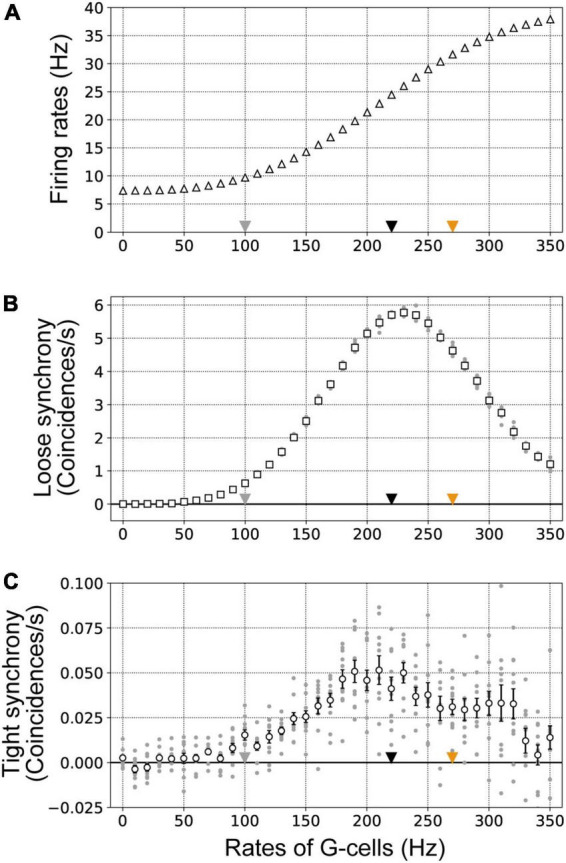
Firing rates **(A)**, loose synchrony **(B)**, and tight synchrony **(C)** of border ownership selective (BOS) model neurons with systematic variation of the mean G-cell firing rate (ν*_*G*_*). ν*_*G*_* was varied in the range of 0–350 Hz in steps of 10 Hz. These data were computed from 10 trials of 50 simulations for each mean ν*_*G*_*. Gray dots show the firing rates, loose synchrony, and tight synchrony for each trial. Gray, black, and orange triangles indicate the firing rates of G-cells used to represent the Unbound-ignored, Bound-ignored, and Bound-attended conditions, respectively. Error bars indicate the standard error. **(A)** The mean firing rates of BOS model neurons are shown by white triangles. **(B)** White squares indicate the mean magnitudes of loose synchrony of BOS model neurons between V2 units. **(C)** The mean magnitudes of tight synchrony for BOS model neurons are shown by white circles.

Figure 7 summarizes the firing rates, loose synchrony, and tight synchrony of BOS model neurons as a function of the firing activity of G-cells. The firing rates of BOS model neurons monotonically increased as G-cells were activated (Figure 7A). In contrast, the magnitude of loose synchrony between BOS model neurons exhibited a non-monotonic modulation pattern, increasing until peaking when the G-cell firing rate was approximately 230 Hz and then decreasing (Figure 7B). These results indicated that, in the disinhibitory network, BOS model neurons in both units are simply activated by feedback signals from G-cells in V4. However, significant activation of common signals to VIP interneurons could induce asynchronous responses between BOS neurons across units, which was consistent with the characteristics observed during physiological modulation of BOS neurons (Martin and von der Heydt, 2015). The plausible mechanisms for inducing contrasting modulation between the firing rate and loose synchrony will be discussed in the Discussion section.

Figure 7C represents the magnitude of tight synchrony of BOS model neurons between V2 units when parametrically varying the frequency of G-cells in our proposed model. The tight synchrony fluctuations of BOS neurons between units were greater than those observed for the firing rate (Figure 7A) and loose synchrony (Figure 7B). In addition, the magnitude of tight synchrony was much smaller than that of loose synchrony, as shown in Figure 7B. However, similar to loose synchrony, a trend was observed for a tight synchrony maximum with G-cell activity of approximately 200 Hz. Interestingly, the magnitude of tight synchrony also decreased beyond a G-cell firing rate of approximately 230 Hz, similar to that of loose synchrony. These results suggested that feedback signals to VIP interneurons might induce tight synchrony in BOS neurons between V2 units over a broad range of feedback firing rates.

### Influence of the synaptic strength between G-cells and V2 units on modulation of border ownership selective model neuron responses

The synaptic strength of excitatory neurons is a critical factor for modulation of the responses and dynamics of neuronal networks (Teramae et al., 2012; Wagatsuma et al., 2016). To investigate the influence of feedback signals from G-cells in detail, we performed simulations using our proposed network model with various synaptic weights for connections from G-cells to VIP model interneurons ($w_{i\,n\,p\,u\,t}^{G-V\,I\,P}$). In these simulations, the firing rates of G-cells were fixed at 220 Hz.

**FIGURE 8:**
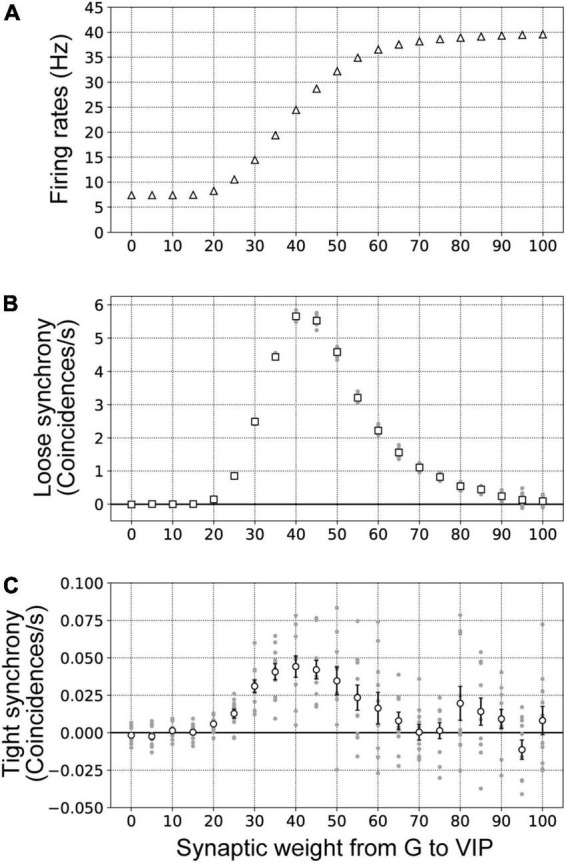
Firing rates **(A)**, loose synchrony **(B)**, and tight synchrony **(C)** for border ownership selective (BOS) model neurons with variation of the synaptic weight of connections from G-cells to vasoactive intestinal polypeptide (VIP) interneurons. The synaptic weight was varied in the range from 0 to 100 in steps of 5. These data were computed from 10 trials of 50 simulations for each synaptic weight. The conventions were the same as those in Figure 7. **(A)** The mean firing rates of BOS model neurons are shown by white triangles. **(B)** White squares indicate the mean magnitudes of loose synchrony of BOS model neurons between V2 units. **(C)** The mean magnitudes of tight synchrony for BOS model neurons are shown by white circles.

Figure 8A presents the firing rates of BOS model neurons with systematic variation of synaptic weights for connections from G-cells to VIP model interneurons ($0\leq w_{i\,n\,p\,u\,t}^{G-V\,I\,P}\leq 100$). The firing rates of BOS model neurons increased when the synaptic weight of feedback signals from V4 was increased. However, a significantly strong synaptic weight of G-cells ($w_{i\,n\,p\,u\,t}^{G-V\,I\,P}>60$) had little influence on the firing rate of BOS model neurons. Loose and tight synchrony of BOS model neurons between V2 units as a function of synaptic weight $w_{i\,n\,p\,u\,t}^{G-V\,I\,P}$ is summarized in Figures 8B,C, respectively. Marked peaks of loose and tight synchrony were observed between BOS model neurons with an intermediate synaptic weight of approximately $w_{i\,n\,p\,u\,t}^{G-V\,I\,P}=40$. However, these peaks decreased with increasing synaptic weight. These monotonic increases in firing rate and non-monotonic modulation of spike synchrony of BOS model neurons with increasing $w_{i\,n\,p\,u\,t}^{G-V\,I\,P}$ were similar to the modulation patterns induced by G-cell activation (Figure 7). These results indicated that, in addition to the spike frequency of common inputs to the disinhibitory network, synaptic strength plays a fundamental role in inducing spike synchrony.

### Influences of the frequency of feedforward inputs representing visual stimuli on modulation of border ownership selective model neuron responses

In our disinhibitory network model, the activation of G-cells in V4 played a critical role in inducing paradoxical attentional modulation of BOS neurons in V2 with regard to their firing rates and spike synchrony. However, previous computational and psychophysical studies have suggested that attentional activation of V1 neurons might underlie the attentional modulation in BOS neurons in V2 (Wagatsuma et al., 2008, 2013). To investigate the influence of feedforward inputs on modulation of the responses of BOS neurons in our disinhibitory network, we performed simulations of the model with feedforward input frequencies of 150 and 250 Hz. In these simulations, we provided 220 Hz signals from G-cells to both V2 units.

**FIGURE 9:**
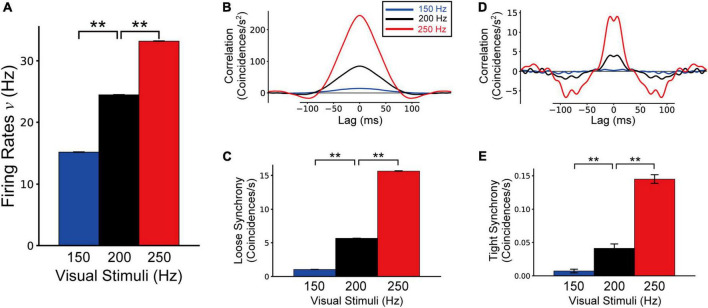
Firing rates **(A)**, loose synchrony **(B,C)**, and tight synchrony **(D,E)** of border ownership selective (BOS) model neurons in response to feedforward inputs of 150, 200, and 250 Hz. These data were computed from 10 trials of 50 simulations for each feedforward input rate. Blue, black, and red bars and lines represent the simulation results for feedforward inputs of 150, 200, and 250 Hz, respectively. Black bars and lines indicating feedforward inputs of 200 Hz are identical to the results for the bound-ignored condition (black bars and lines in Figures 4–6). Asterisks indicate significant differences between conditions (**p < 0.01, *t*-test).

We show the firing rates, loose synchrony, and tight synchrony of BOS model neurons under feedforward input frequencies of 150 and 250 Hz in Figure 9. We also present the simulation results of the Bound-ignored condition (200 Hz feedforward inputs) for comparison. Firing rates of BOS neurons were significantly activated with the increase in the frequency of feedforward inputs (Figure 9A; *t*-test, *p* < 0.01). Additionally, the magnitudes of loose (Figures 9B,C) and tight synchrony (Figures 9D,E) were also significantly enhanced as the feedforward inputs were activated (*t*-test for loose and tight synchrony, *p* < 0.01). These concomitant enhancements of firing rates and spike synchrony of BOS model neurons were distinct from the attention-induced paradoxical modulation of physiological BOS neurons in terms of their firing rates and spike synchrony (Martin and von der Heydt, 2015). In addition, SOM inhibitory model interneurons also exhibited concomitant enhancements of firing rates and spike synchrony as feedforward input frequencies increased (Supplementary Figure 4).

## Discussion

In the present study, to investigate the neural mechanism underlying the grouping-structure-induced and attention-induced modulation of firing rates and spike synchrony of BOS neurons (Martin and von der Heydt, 2015), we proposed a disinhibitory network model including not only BOS model neurons but also two subtypes of inhibitory interneurons (Buia and Tiesinga, 2008). In our model, the functional unit for border ownership selectivity in the V2 region (V2 unit) consisted of an excitatory BOS model neuron and SOM and VIP inhibitory model interneurons (Figure 3). In this V2 unit, the BOS neuron and SOM interneuron interacted with each other. Inhibitory projections from the VIP interneuron to the SOM model interneuron were present within the unit. Additionally, G-cells in V4 project the feedback signals to VIP interneurons in both V2 units as common inputs. In our model, G-cells represent the grouping structure and mediate selective attention. Simulations of our proposed network model indicated that the firing rates of BOS model neurons increased with G-cell activation. However, significant activation of G-cells reduced the spike synchrony of BOS model neurons between V2 units, which was consistent with the physiological results of BOS neurons in intermediate-level visual areas (Martin and von der Heydt, 2015).

### Mechanism by which the disinhibitory network modulated the activity and spike synchrony of excitatory neurons

Simulations of our disinhibitory network in which VIP interneurons were connected to SOM interneurons indicated that significant activation of feedback signals induced a paradoxical decrease in spike synchrony with increasing firing rate in BOS model neurons, which is consistent with the physiological characteristics of attentional modulation of BOS neurons in intermediate visual areas (Qiu et al., 2007; Martin and von der Heydt, 2015). The activities of two subtypes of inhibitory interneurons, SOM and VIP, played an important role in modulating the responses of BOS neurons. The firing rates of VIP and SOM model inhibitory interneurons as a function of the firing activity of G-cells are shown in Supplementary Figures 3A,B, respectively. In our model, significant feedback signals from G-cells markedly activated the VIP model interneurons in two V2 units at the same time (Supplementary Figure 3A), which inhibited SOM model interneuron responses in both units (Supplementary Figure 3B). Consequently, inhibitory signals from SOM to BOS model neurons might decrease as G-cell activation increases. As a result, BOS model neuron responses were increased as the firing rates of G-cells increased (Figure 7A). In contrast, the frequency of feedforward inputs representing visual stimuli was identical irrespective of stimulus conditions. A reduction of inhibitory signals to BOS model neurons (Supplementary Figure 3B and *I*_*SOM*_ in Eq. 1) might underlie the activation of BOS neurons in both units.

Supplementary Figures 3C,D summarize loose synchrony of VIP and SOM model interneurons, respectively, as a function of the G-cell firing rates. A previous computational study reported that more frequent activation of common inputs mediated by AMPA synaptic receptors increased the magnitude of spike synchrony between pairs of postsynaptic neurons (Wagatsuma et al., 2016). Similarly, in our network model, spike synchrony of VIP model interneurons between V2 units was monotonically strengthened according to the G-cell firing rate (Supplementary Figures 1A, 2C), which induced the synchronized activation of SOM model interneurons between units under the Bound-ignored condition (Figure 3B and Supplementary Figure 1B). In addition, the synchrony of SOM interneurons between units may reset the membrane potentials of BOS model neurons across units at approximately the same time. Synchronized responses of SOM model interneurons might contribute to the generation of spike synchrony between BOS neurons under the Bound-ignored condition in our network model.

In contrast to the Bound-ignored condition, under the Bound-attended condition (Figure 3A), SOM model interneuron activity was significantly inhibited by activation of VIP model interneurons (Figure 4B and Supplementary Figures 3A,B) because of selective attention mediated by G-cells. This inhibition of SOM interneurons underlies the attentional enhancement of BOS model neuron responses in our network model. In our proposed network, BOS model neurons integrated the inhibitory signals from the SOM model interneuron in the same unit and the excitatory feedforward inputs representing visual stimuli given by the Poisson spike trains (Figure 3). Under the Bound-attended condition, as a result of significant inhibition of the SOM model interneuron, the feedforward inputs acted as dominant inputs to the BOS model neurons, which might induce more random spikes of the BOS model neurons and decrease the spike synchrony of these model neurons between units. In addition, significant activation of G-cells not only inhibited the responses of SOM interneurons (Supplementary Figure 3B) but also decreased the spike synchrony of SOM neurons between V2 units (Supplementary Figure 3D). The interactions between the inhibition of activity and the decrease in spike synchrony of SOM interneurons via significant G-cell activation might induce dyssynchrony of BOS model neurons between units. The disinhibition mediated by the inhibition of SOM interneurons by VIP interneurons is a possible mechanism for the paradoxical decrease in synchrony with attentional activation of BOS neurons. However, the mechanism of the disinhibitory network model for the attention-induced paradoxical modulation of BOS neurons in terms of their firing rates and spike synchrony seems to be distinct from that of the previous models based on NMDA-mediated feedback signals (Wagatsuma et al., 2016, 2021).

Spike synchrony of VIP inhibitory model interneurons monotonically increased with G-cell activation (Supplementary Figure 3C). These synchronized inhibitory signals might induce spike synchrony of SOM inhibitory model interneurons between two units. In contrast, spike synchrony of SOM inhibitory model interneurons between two units showed a non-monotonic modulation pattern, increasing until peaking when the G-cell firing rate was approximately 150 Hz and then decreasing (Supplementary Figure 3D). The synchronized inhibitory signals from VIP model interneurons decreased the membrane potentials in SOM model interneurons in two different units at the same time, which might have generated the synchronized responses of SOM interneurons between units. However, significantly activated and synchronized VIP model interneurons might have preserved low membrane potentials in SOM interneurons, thus remaining below the spike threshold, which prevented SOM interneurons from generating spikes and reduced the synchronized activities for these interneurons between units. The non-monotonic modulation of spike synchrony for SOM model interneurons seemed to arise from interactions between synchronized signals from VIP interneurons and the firing frequency of SOM interneurons.

In contrast to G-cell activation, the firing rates and spike synchrony of SOM inhibitory model interneurons were increased with increasing frequency of visual inputs (Supplementary Figure 4). In addition, there was a significant increase in the spike synchrony of BOS model interneurons between units as the visual inputs were activated (Figure 9). These results suggested the important role of SOM interneuron synchrony in inducing BOS neuron synchrony between units.

The functional roles of spike synchrony of neuron pairs for perceiving the visual scene have been investigated by various studies (Niebur and Koch, 1994; Steinmetz et al., 2000; Dong et al., 2008). However, our computational model suggested that the attention-induced paradoxical modulation of BOS neurons between their firing rates and spike synchrony is epiphenomenally induced by the disinhibitory network consisting of SOM and VIP inhibitory interneurons in addition to excitatory BOS neurons. Further studies are needed to delve deeper into the functional role of spike synchrony between BOS neurons.

### Mechanism of attentional modulation of responses in border ownership selective neurons

In this study, BOS model neuron activity was modulated by selective attention through inhibitory connections from SOM to VIP model interneurons (Figure 4A). In addition, VIP model interneuron responses were determined by common feedback signals from G-cells to two V2 units. To simplify our simulations, we represented all excitatory external inputs including feedback signals as AMPA-type synaptic currents. However, physiological evidence indicates that fast driving of AMPA receptors provides feedforward inputs to V1, whereas the feedback signals mediated by NMDA-type currents underlie figure-ground modulation (Self et al., 2012; Herrero et al., 2013). Furthermore, our previous computational studies indicated the contribution of direct projections of modulatory common feedback signals mediated by NMDA-type synapses to the attentional modulation of firing rates and spike synchrony in BOS neurons (Wagatsuma et al., 2016, 2021). However, in contrast to the current study, these previous models did not include any inhibitory interneuron subtypes. A network model based on various model interneuron subtypes and detailed synaptic models of external inputs may be necessary to clearly understand the mechanism of attentional modulation of BOS neurons.

### Relationship between the current model and previous studies based on n-methyl-d-aspartate-mediated feedback signals for attentional modulation of border ownership selective neurons

Selective attention increased the firing frequency of BOS neurons but decreased spike synchrony among these neurons during the coding of a common object (Martin and von der Heydt, 2015), which was reproduced by our current disinhibitory network model (Figures 4–6). However, other computational models based on the grouping hypothesis also reproduced the attention-induced paradoxical modulation of BOS neurons in terms of their firing rates and spike synchrony (Wagatsuma et al., 2016, 2021). Feedback signals from G-cells in V4 to the V2 area were common to our current and these previous models. As previously discussed, in our current study, disinhibitory connections from VIP to SOM interneurons and activation of VIP interneurons by the feedback signals underlie the attentional modulation of BOS model neurons. In contrast, in these previous models (Wagatsuma et al., 2016, 2021), BOS model neurons in different V2 units received modulatory feedback signals mediated by synaptic currents through NMDA receptors. These modulatory influences and the slow timescale of NMDA-mediated excitation play an important role for paradoxical attentional modulation in BOS model neurons. The two different mechanisms of our current model and these previous modeling studies were proposed based on physiologically plausible evidence (Self et al., 2012; Pi et al., 2013; Pfeffer, 2014; Zhang et al., 2014). However, the simulation results of these disinhibitory network and NMDA models were slightly different in the characteristics of the tight correlation of BOS neurons between two units. For the tight correlation of the NMDA model, marked peaks were present at a lag of zero in the Bound-ignored and Bound-attended conditions (Figure 6 in Wagatsuma et al., 2016 and Figure 5 in Wagatsuma et al., 2021). In contrast, in the Bound-ignored and Bound-attended conditions of our disinhibition model, we found marked peaks of tight correlation not at but around a lag of zero (Figure 6). These results implied the possibility that marked common excitatory inputs to two neurons might play an important role in inducing the tight correlation between them at a lag of zero.

Both our current disinhibitory network model and previous NMDA models (Wagatsuma et al., 2016, 2021) reproduced the attention-induced paradoxical modulation of physiological BOS neurons in terms of their firing frequency and spike synchrony (Martin and von der Heydt, 2015). However, the detailed neuronal mechanisms for attentional modulation in physiological BOS neurons are still unknown. In previous physiological experiments, the activities of excitatory neurons have been recorded while optically activating a specific subtype of inhibitory interneuron to examine the mechanism of orientation tuning in rodent V1 (Atallah et al., 2011; Wilson et al., 2012). The methods used in these experiments might be applied to understand the neuronal mechanism of attentional modulation in physiological BOS neurons. However, in these physiological experiments, neuronal responses were not recorded from monkeys but were obtained from rodents. In addition, although another physiological study reported figure-ground modulation in the mouse V1 (Schnabel et al., 2018), it seems to be difficult to examine the mechanism of selective attention in physiological experiments using rodents. Further physiological evidence is necessary to understand the detailed neuronal mechanism of attentional modulation in BOS neurons.

### Influences of synaptic decay and the membrane time constant on the spike synchrony between border ownership selective neurons

In the current study, the widths of the loose correlation of BOS model neurons between V2 units (Figure 5B) had similar levels to those observed in physiological BOS neurons (Figure 5A; Martin and von der Heydt, 2015). Interestingly, these broad widths of the loose synchrony of BOS model neurons have been observed in the simulation results of previous NMDA models (Wagatsuma et al., 2016, 2021). In contrast, the loose correlation of VIP model neurons (Supplementary Figure 1A) was much sharper than that of BOS model neurons. The differences in the widths of loose synchrony seemed to arise from the postsynaptic decay time constant. In our disinhibition network model, the common feedback signals from the G-cells to both V2 units might have induced spike synchrony between VIP model neurons, which was mediated by the AMPA-type synaptic currents of τ_*Input*_ = 2.0 ms for the postsynaptic decay time constant (see also Eq. 9). In contrast, in previous NMDA models (Wagatsuma et al., 2016, 2021), the common modulatory feedback signals from G-cells were mediated by NMDA synaptic receptors with slow temporal dynamics for the 80 ms of the postsynaptic decay time constant (Wang, 1999). In the current study, we applied 13 ms of postsynaptic decay to inhibitory connections from VIP to SOM and from SOM to BOS model neurons (Pfeffer et al., 2013; Hoffmann et al., 2015; Lee et al., 2018; see also Table 2). A slower postsynaptic decay might induce spike synchrony with a broader curve. Additional simulations with various parameters of common signals to two V2 units may provide insight into the mechanisms of neuronal synchrony.

Under our selected parameters for the disinhibition network model, we observed the peak of loose synchrony between model BOS neurons when these neurons were activated to 25 Hz (Figures 7, 8). In the spike-field coherence of the physiological BOS neurons, a peak was also observed at approximately 25 Hz for the Bound-attended condition (Figure 7 in Martin and von der Heydt, 2015). Interestingly, this peak of BOS neurons at 25 Hz was within the beta band activity in the range between 20 and 30 Hz, which might be induced by the responses of inhibitory SOM interneurons (Chen et al., 2017; Veit et al., 2017; Wagatsuma et al., 2022). In contrast, the activity of PV interneurons, the largest inhibitory population, preferentially generated faster synchronized responses such as gamma band activity (Chen et al., 2017; Wagatsuma et al., 2022). The membrane time constant (τ_*m*_ in Eqs. 1, 2, and 3) is a possible parameter that can induce distinct frequency band activities in excitatory neurons. The τ_*m*_ of PV interneurons was much faster than that of SOM interneurons (Neske et al., 2015). The firing rates that induce synchronized neuronal responses might be determined by the interaction between τ_*m*_ and the postsynaptic decay time constant. It is possible that SOM interneurons contributed to the enhancement of the synchronized responses near the beta band activity for excitatory neurons.

### Limitations of the current model and comparison with previous models

Martin and von der Heydt (2015) investigated the physiological characteristics of attentional modulation in BOS neurons for consistent and inconsistent pairs: if the preferred sides of both members of a neuron pair were consistent with the common object (Figure 2), the pair was called “consistent” for this object; all other pairs were called “inconsistent” (Wagatsuma et al., 2016, 2021). However, the current study was restricted to investigation of the behaviors of a consistent pair of BOS neurons. Extension of the disinhibitory network model is necessary to enhance our understanding of the mechanisms of figure-ground segregation. However, additional BOS model neurons or V2 units sharing a common CRF and with distinct border ownership selectivity are needed to understand the mechanism of modulation of inconsistent BOS neuron pairs. We might also add interactions within common CRFs between BOS model neurons or V2 units. Furthermore, additional subtypes of PV interneurons should be introduced to the extended model because a previous study physiologically reported the contribution of PV interneurons to the neuronal selectivity in V1 (Lee et al., 2012). Further discussions of the suitable structure for inconsistent pairs of BOS neurons seem to be necessary to extend the disinhibitory network model.

The previous disinhibitory network model proposed by Buia and Tiesinga (2008) consisted of an excitatory neuron class and two subtypes of inhibitory interneurons. In this previous study, to reproduce the neural modulation observed in the V2 region (Reynolds et al., 1999), the two units had common receptive fields and interacted with each other through inter-unit connections from excitatory neurons to the specific inhibitory interneuron subtypes. In contrast to this model, there were no direct connections between two V2 units in our present model because the CRF locations for these units did not retinotopically overlap (Figure 3). Such interactions among neurons sharing their CRFs might be necessary to model the behaviors of inconsistent pairs of BOS neurons, as described in the previous paragraph.

To investigate the neural mechanism of figure-ground segregation, we developed a microcircuit model consisting of an excitatory BOS neuron and two subtypes (SOM and VIP) of inhibitory interneurons (Figure 3). These distinct subtypes of interneurons seemed to work differently in regulating the neural responses for visual perception and attentional modulation. However, to simplify the current model, we did not introduce the PV-expressing subtype of interneuron, which comprises the largest population of inhibitory interneurons in the superficial layer of the primary visual area (Rudy et al., 2011; Pfeffer et al., 2013; Lee et al., 2017). A microcircuit model including three subtypes of inhibitory interneurons will provide insight into the neural mechanisms of figure-ground segregation.

## Data availability statement

The raw data supporting the conclusions of this article will be made available by the authors, without undue reservation.

## Author contributions

NW: design, methodology, analysis, software, and writing—original draft. NW and SN: funding acquisition. SN: writing—review and editing. HS: software and computational data acquisition. All authors contributed to the article and approved the submitted version.
