# Supplementary material for: Disinhibitory circuit mediated by connections from vasoactive intestinal polypeptide to somatostatin interneurons underlies the paradoxical decrease in spike synchrony with increased border ownership selective neuron firing rate
Source: Front Comput Neurosci. 2022 Nov 4;16:988715. doi: 10.3389/fncom.2022.988715 (PMC9672816; doi:10.3389/fncom.2022.988715)
Supplement: Supplementary file 1 [file Data_Sheet_1.pdf]

Supplementary Material

1. Supplementary Figures

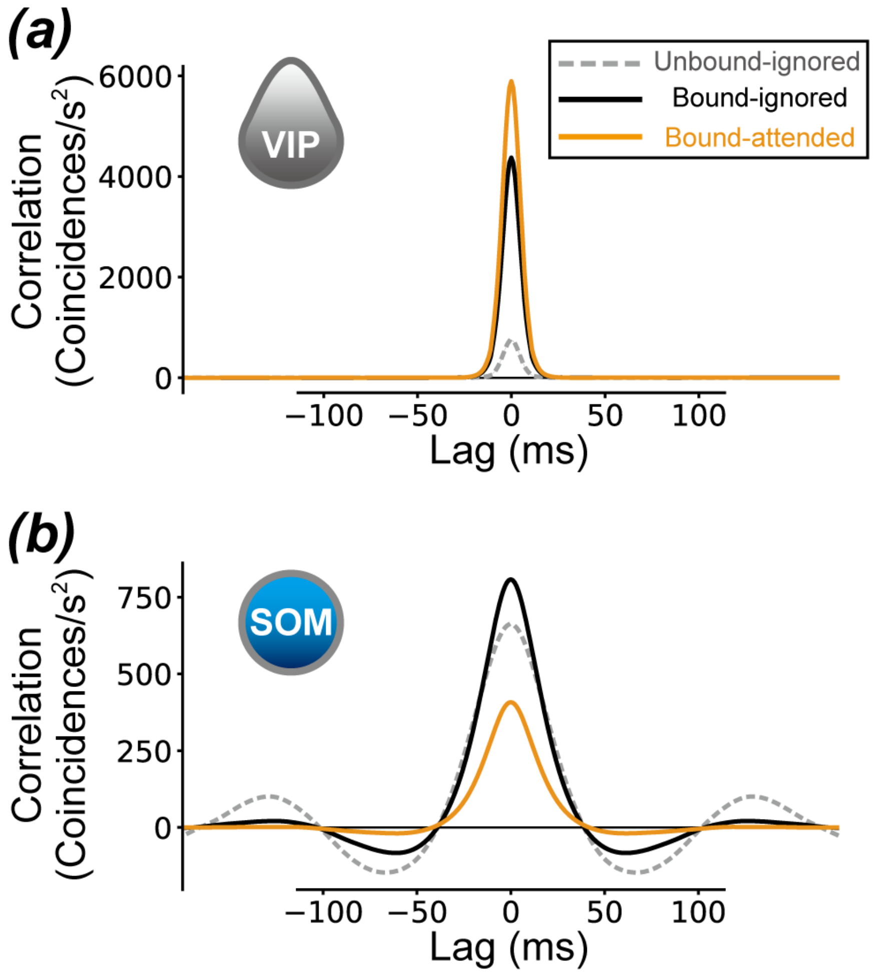

Supplementary Figure 1. (a) Loose correlations of VIP model interneurons between V2 units. The gray dashed, black slid, and orange solid lines represent the spike correlation for the Unbound-ignored, Bound-ignored, and Bound-attended conditions, respectively. The strengths of loose correlations for VIP model interneurons strongly depended on the firing rates of G-cell. In addition, the loose correlation of VIP model interneurons was much sharper than that of BOS model neurons (Figure 5(b)). (b) Loose correlations of SOM model interneurons between V2 units. The conventions were the same as those in the panel (a). In contrast to the loose correlation for VIP model interneurons, the Bound-attended condition induced the weakest peak of loose correlation of SOM model interneurons between V2 units.

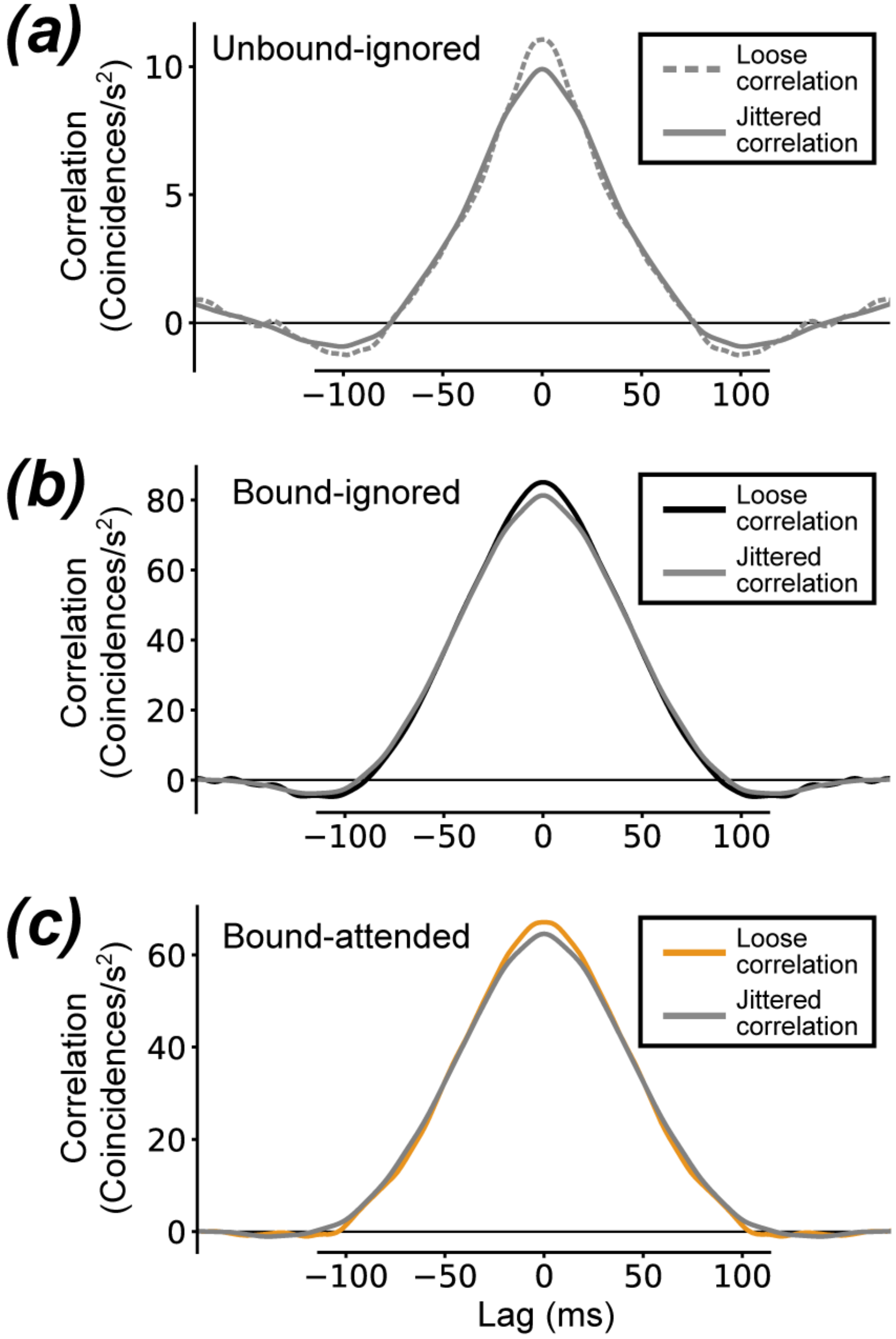

Supplementary Figure 2. Jittered correlations (gray solid lines) for (a) Unbound-ignored, (b) Bound-ignored, and (c) Bound-attended conditions. The curves of jittered correlations around a lag of zero are sharper compared to those of loose correlations.

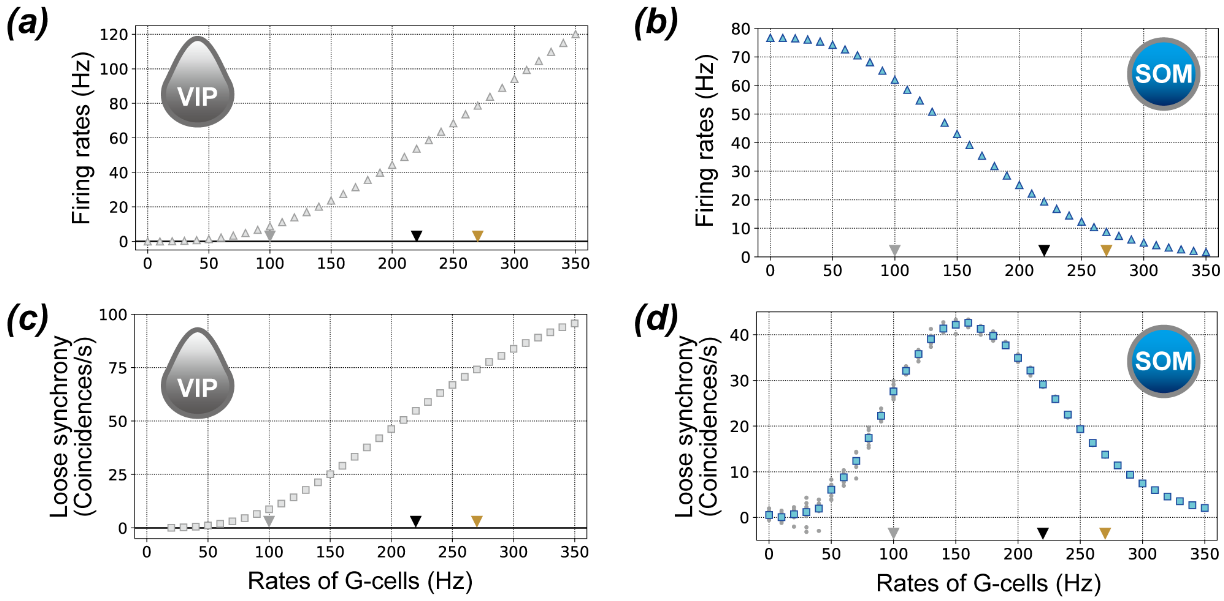

Supplementary Figure 3. (a) Firing rates of VIP model interneuron with systematic variation of the mean G-cell firing rate (v_G). (b) Firing rates of SOM model interneuron with systematic variation of the mean v_G. (c) Loose synchrony of VIP interneurons between different V2 units as a function of the mean v_G. (d) Loose synchrony of SOM interneurons between different V2 units as a function of the mean v_G. Gray dots show the firing rates and loose synchrony for each trial. Gray, black, and orange triangles indicate the firing rates of G-cells used to represent the Unbound-ignored, Bound-ignored, and Bound-attended conditions, respectively. Firing rates (a) and loose synchrony of VIP model interneurons (c) are monotonically increased as the increase of v_G. By contrast, firing rates of SOM model interneurons (b) are monotonically decreased with the activation of G-cells. In addition, the loose synchrony between SOM model interneurons exhibited a nonmonotonic modulation pattern, increasing until peaking when v_G was approximately 160 Hz and then decreasing. v_G inducing the peak of loose synchrony between SOM model interneurons was lower compared to the case of the peak for BOS model neurons (Figure 7(b)).

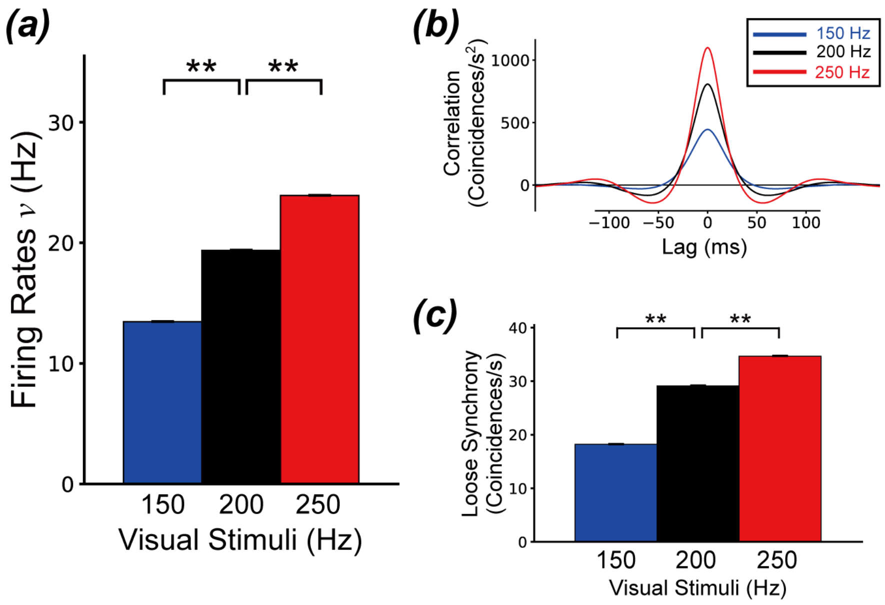

Supplementary Figure 4. Firing rates (a), loose correlation (b) and loose synchrony (c) of SOM model neurons in response to feedforward inputs of 150, 200, and 250 Hz. These data were computed from 10 trials of 50 simulations for each feedforward input rate. Blue, black, and red bars and lines represent the simulation results for feedforward inputs of 150 Hz, 200 Hz, and 250 Hz, respectively. Black bars and lines indicating feedforward inputs of 200 Hz are identical to the results for the bound-ignored condition (black bars and lines in Figures 4, 5, and 6). Asterisks indicate significant differences between conditions (** $p < 0.01$, t-test).
